# Comprehensive bioinformatics and machine learning analysis identify VCAN as a novel biomarker of hepatitis B virus-related liver fibrosis

**DOI:** 10.3389/fmolb.2022.1010160

**Published:** 2022-10-07

**Authors:** Mengqin Yuan, Xue Hu, Lichao Yao, Pingji Liu, Yingan Jiang, Lanjuan Li

**Affiliations:** ^1^ Department of Infectious Diseases, Renmin Hospital of Wuhan University, Wuhan, Hubei, China; ^2^ State Key Laboratory for Diagnosis and Treatment of Infectious Diseases, National Clinical Research Centre for Infectious Diseases, Collaborative Innovation Centre for Diagnosis and Treatment of Infectious Diseases, The First Affiliated Hospital, Zhejiang University School of Medicine, Hangzhou, Zhejiang, China

**Keywords:** hepatitis B virus-related liver fibrosis, diagnostic biomarker, bioinformatic analysis, machine-learning strategies, immune cell infiltration

## Abstract

Hepatitis B virus (HBV) infection remains the leading cause of liver fibrosis (LF) worldwide, especially in China. Identification of decisive diagnostic biomarkers for HBV-associated liver fibrosis (HBV-LF) is required to prevent chronic hepatitis B (CHB) from progressing to liver cancer and to more effectively select the best treatment strategy. We obtained 43 samples from CHB patients without LF and 81 samples from CHB patients with LF (GSE84044 dataset). Among these, 173 differentially expressed genes (DEGs) were identified. Functional analysis revealed that these DEGs predominantly participated in immune-, extracellular matrix-, and metabolism-related processes. Subsequently, we integrated four algorithms (LASSO regression, SVM-RFE, RF, and WGCNA) to determine diagnostic biomarkers for HBV-LF. These analyses and receive operating characteristic curves identified the genes for phosphatidic acid phosphatase type 2C (PPAP2C) and versican (VCAN) as potentially valuable diagnostic biomarkers for HBV-LF. Single-sample gene set enrichment analysis (ssGSEA) further confirmed the immune landscape of HBV-LF. The two diagnostic biomarkers also significantly correlated with infiltrating immune cells. The potential regulatory mechanisms of VCAN underlying the occurrence and development of HBV-LF were also analyzed. These collective findings implicate VCAN as a novel diagnostic biomarker for HBV-LF, and infiltration of immune cells may critically contribute to the occurrence and development of HBV-LF.

## Introduction

Hepatitis B virus (HBV) infection remains the leading cause of acute and chronic liver disease and is associated with high morbidity and mortality. Approximately 20%–30% of chronic hepatitis B (CHB) patients may develop progressive liver fibrosis (LF), which leads to an increased risk of cirrhosis and liver cancer (Vittal and Ghany, 2019). However, the clinical manifestations and symptoms of early HBV-LF are nonspecific. Prevention and early diagnosis are the most effective approaches to improving the prognosis of patients with HBV-LF. Hepatic biopsy is the “gold standard” for diagnosing and staging liver diseases (Nakajima et al., 2020; Virarkar et al., 2021). It is worth noting that the biopsy procedure is invasive and the risk of serious complications is as high as 1%. Furthermore, heterogeneous distribution of fibrosis can lead to sampling errors in liver biopsy. Ultrasonography is the first choice for screening LF because it is non-invasive, non-radioactive and inexpensive (Lurie et al., 2015). Nevertheless, the disadvantage of ultrasonography is its’ low sensitivity in assessing LF. Serum markers/indices of LF, including the fibrosis-4 index, Aspartateaminotransferase-to-Platelet Ratio Index and Lok index, have high clinical and diagnostic values (Agbim and Asrani, 2019). However, identifying novel biomarkers is crucial for improving the early diagnosis of LF.

In recent years, high-throughput sequencing technologies have provided powerful means for investigating the mechanisms and characteristics of liver disease (Oh et al., 2020; Torres et al., 2020). For example, Gong et al. identified that compared with normal liver, there is a unique gene expression pattern for immune response, necroptosis, and apoptosis in liver samples of HBV-related acute liver failure (Gong et al., 2022). Based on bioinformatics analyses, many pivotal genes that are essential for liver disease have been identified. Wang et al. (2021) identified four genes (CYP26A1, FAM110C, SMYD3, and ZG16) that were expected to be novel diagnostic and prognostic targets for HBV-related hepatocellular carcinoma (HCC). However, many researchers have focused on LF and HBV-LF has rarely been studied. Consequently, additional studies are required to identify novel diagnostic biomarkers to differentiate the diagnoses and clarify the underlying molecular mechanisms of HBV-LF.

In this study, the matrix file of HBV-LF was obtained from the Gene Expression Omnibus (GEO) database, and differential expression of genes was analyzed. Subsequently, three machine learning strategies and the weighted gene co-expression network analysis (WGCNA) algorithm were integrated to identify the diagnostic biomarkers for HBV-LF. Next, single-sample gene set enrichment analysis (ssGSEA) was conducted to assess the differences in 28 immune cell subsets between HBV-LF and CHB samples. Moreover, the relationship between biomarkers and immune cells was studied to provide more insights into the molecular mechanism involved in the progression of HBV-LF.

## Materials and methods

### Data collection

The matrix file of the GSE84044 dataset was obtained from the GEO database (http://www.ncbi.nlm.nih.gov/geo/), which included 43 liver biopsy samples from CHB patients without LF and 81 liver biopsy samples from CHB patients with different stages of LF. All these patients were diagnosed according to the Asian Pacific Association for the Study of the Liver (APASL) criteria (Wang et al., 2017). We also obtained corresponding clinical information from the 124 patients (Table 1). The clinical information included gender, age, histological stage of fibrosis, inflammation grade, and two biochemical markers (ALT and AST). To test the diagnostic efficacy of the diagnostic biomarkers, the matrix file of 10 CHB and 10 HBV-LF samples from the GSE114783 dataset were downloaded as a verification cohort.

**TABLE 1:** Clinical information of patients in the GSE84044.

Covariates	Total (n = 124)
**Age**	
<40	60 (48.39)
≥40	64 (51.61)
**Gender**	
Male	88 (70.97)
Female	36 (29.03)
**Grade of inflammation**	
0	37 (29.84)
1	33 (26.61)
2	34 (27.42)
3	15 (12.10)
4	5 (4.03)
**Histological stage of fibrosis**	
0	43 (34.68)
1	20 (16.13)
2	33 (26.61)
3	18 (14.52)
4	10 (8.06)
**ALT**	121.53 ± 191.53
**AST**	74.91 ± 97.06

### Differential expression analysis

R package “Limma” was performed on the dataset of GSE84044 to identify differentially expressed genes (DEGs; |log_2_FC| > 0.585 and FDR <0.05). A protein–protein interaction (PPI) network based on theses DEGs was then constructed using a Search Tool for the Retrieval of Interacting Genes (STRING) database (https://string-db.org/).

### Functional enrichment analysis

To assess the underlying biological functions and signaling pathways of DEGs, Gene Ontology (GO) and Kyoto Encyclopedia of Genes and genomes (KEGG) analyses were conducted using the “clusterProfiler” R package. To evaluate the DEGs-related diseases, we employed R package “DOSE” for disease ontology (DO) analysis. The “clusterprofiler” R package was used for gene set enrichment analysis (GSEA) to clarify further the functional pathways of significant enrichment (FDR <0.25, NOM *p* < 0.05).

### Screening and verification of diagnostic biomarkers

The least absolute shrinkage and selection operator (LASSO) logistic regression, support vector machine recursive feature elimination (SVM-RFE), random forests (RF), and WGCNA were utilized to identify diagnostic biomarkers. R package “glmnet” were applied for LASSO logistic regression, which was performed by 10-fold cross-validation to adjust the optimal penalty parameter *λ*. R packages “e1071” and “caret” for the SVM-RFE algorithm were used to calculate the point with the smallest cross-validation error, so as to screen diagnostic biomarkers. The RF algorithm was applied using the R package “randomForest” to identify genes that could distinguish HBV-LF from CHB patients with a filter condition of relative importance >0.2. We also adopted WGCNA analysis with the “WGCNA” R package. By calculating Pearson correlation coefficient between the eigengenes of each module and the disease state, modules with the most significant correlation with HBV-LF was determined (*p* < 0.05).

We then examined whether the overlapping genes identified using the four algorithms could be used as diagnostic biomarkers. Receiver operating characteristic (ROC) curve analyses were conducted to evaluate the sensitivity and specificity of these diagnostic biomarkers in distinguishing HBV-LF values patients. We further calculated the area under the curve (AUC) to evaluate the reliability of distinguishing HBV-LF from CHB samples.

### Evaluation of immune cell infiltration

Relative infiltration levels of 28 immune cell subsets in the GSE84044 dataset were quantified using the ssGSEA algorithm. R package “corrplot” was used to visualize the correlation between the 28 types of infiltrating immune cells. R package “vioplot” was executed to draw violin plots to demonstrate the different infiltration levels of 28 kinds of immune cells. Furthermore, LASSO logistic regression investigation was conducted using the “glmnet” R package to identify the immune cells with significant differential infiltration between HBV-LF and CHB. The Spearman rank correlation test was used to further evaluate the relationship between these immune cells and diagnostic biomarkers, and then visualized by the “ggplot2” R package. The criteria for significant association between diagnostic biomarkers and significantly differential immune cells were set as R > 0.50 and *p* < 0.001.

### Validation of Versican (VCAN) expression and construction of the TF–VCAN–miRNA regulatory network

The VCAN expression was further verified using the GSE114783 dataset. Representative immunohistochemical staining images of VCAN in normal liver tissue and liver cancer tissue were downloaded from the human protein atlas (HPA) database (https://www.proteinatlas.org/). We identified the top 100 genes of VCAN with similar expression patterns from the Gene Expression Profiling Interactive Analysis (GEPIA) database (http://gepia.cancer-pku.cn/) (Tang et al., 2017). R package “clusterProfiler” was performed to study the biological functions and signal pathways of these similar/interactive genes of VCAN. To further investigate the regulatory mechanisms of VCAN, the ENCORI (https://starbase.sysu.edu.cn/index.php), miRWalk (http://mirwalk.umm.uni-heidelberg.de/) and miRDB (http://www.mirdb.org/) databases were performed to predict the miRNA that targeting VCAN. Furthermore, the transcriptional regulatory relationships unravelled by the sentence-based text-mining (TRRUST) database (http://www.grnpedia.org/trrust/) were applied to predict the transcription factors (TFs) that regulate VCAN. Subsequently, the TF-VCAN-miRNA regulatory network was visualized using Cytoscape software.

## Results

### Identification of DEGs

**FIGURE 1:**
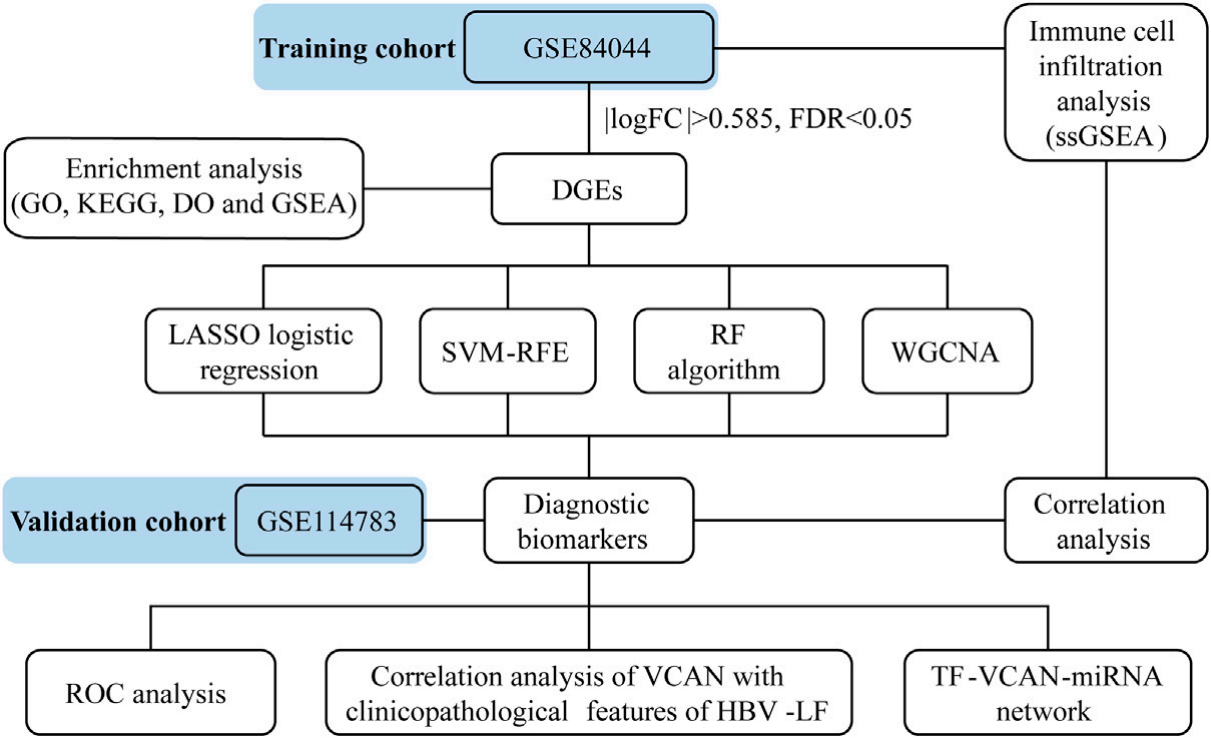
The Flow chart of this research.

**FIGURE 2:**
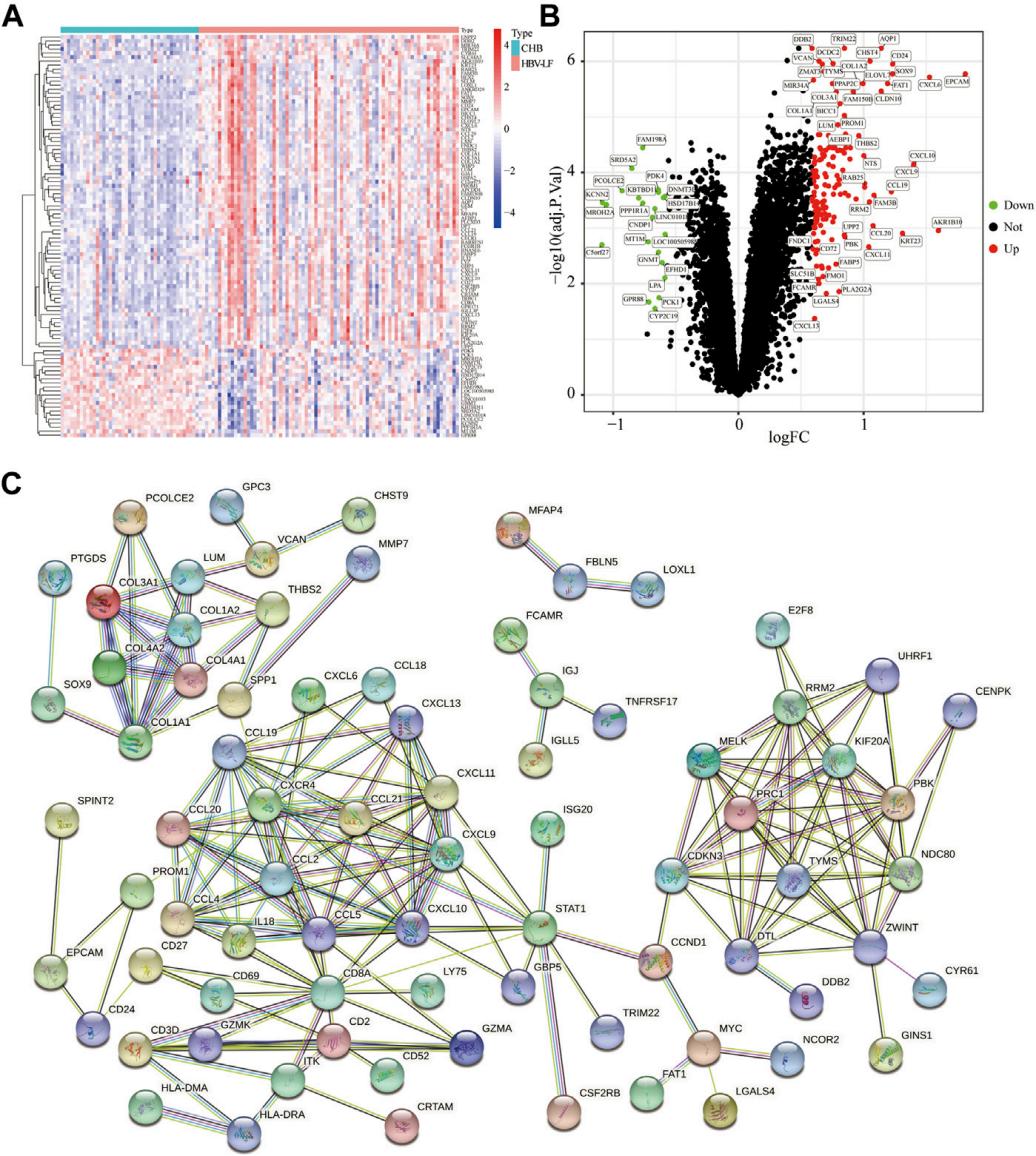
Identification of DEGs between the HBV-LF and CHB samples. **(A)** The heatmap of the DEGs. **(B)** The volcano map of DEGs. **(C)** The PPI network of DEGs.

Figure 1 depicted the flow chart of the research. To identify the DEGs between HBV-LF and CHB samples, we comprehensively studied the expression matrix of the GSE84044 dataset. A total of 173 genes (|log_2_FC| > 0.585, FDR <0.05) were differently expressed. Of these, 22 genes were down-regulated and 151 genes were up-regulated (Figures 2A,B; Supplementary Table S1). Furthermore, we constructed a PPI network to clarify the protein-protein interactions of these DEGs. The minimum interactive score was set to 0.70 to ensure accuracy. As depicted in Figure 2C, the PPI network contained 166 nodes and 206 edges.

### Functional enrichment analysis

**FIGURE 3:**
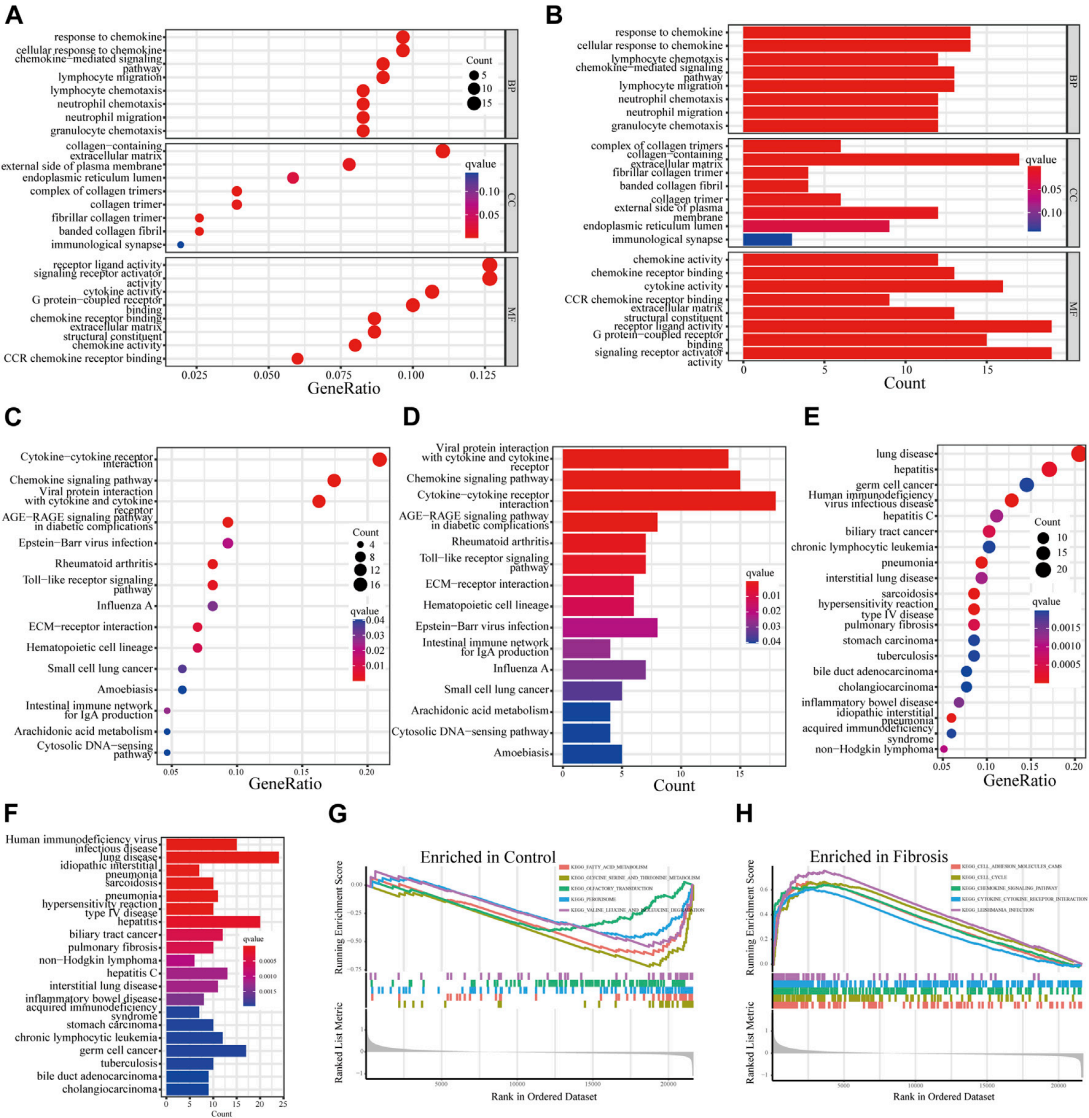
Functional enrichment analysis of DEGs. **(A,B)** GO analyses of DEGs. **(C,D)** KEGG analyses of DEGs. **(E,F)** DO analysis of DEGs. **(G,H)** GSEA analysis.

GO and KEGG analyses were carried out to further illustrate the biological processes and pathways of these DEGs. The GO enrichment analysis results were shown in Figures 3A,B. The biological processes (BP) of DEGs demonstrated that they were involved in response to chemokine and chemokine-mediated signaling pathways. With regard to molecular functions (MF) and cellular components (CC), these DEGs were primarily involved in collagen-containing extracellular matrix (ECM), complex of collagen trimers, cytokine activity, and signaling receptor activator activity. KEGG analysis depicted that these DEGs were mainly involved in the chemokine signaling pathway, cytokine-cytokine receptor interaction, Toll-like receptor signaling pathway and ECM-receptor interaction (Figures 3C,D). We then conducted a DO enrichment analysis to elucidate the role of DEGs in diseases. The result showed that DEGs were significantly related to hepatitis, interstitial lung disease, hepatitis C and other diseases (Figures 3E,F). Moreover, GSEA analyses were conducted to further understand the biological pathways involved in the pathological progress of HBV-LF (Figures 3G,H). The KEGG pathways of GSEA suggested that fatty acid metabolism, threonine metabolism, glycine serine, chemokine signaling pathway, cell adhesion molecules cams, and cytokine-cytokine receptor interaction were significantly enriched in the HBV-LF.

### Identification and verification of diagnostic biomarkers

**FIGURE 4:**
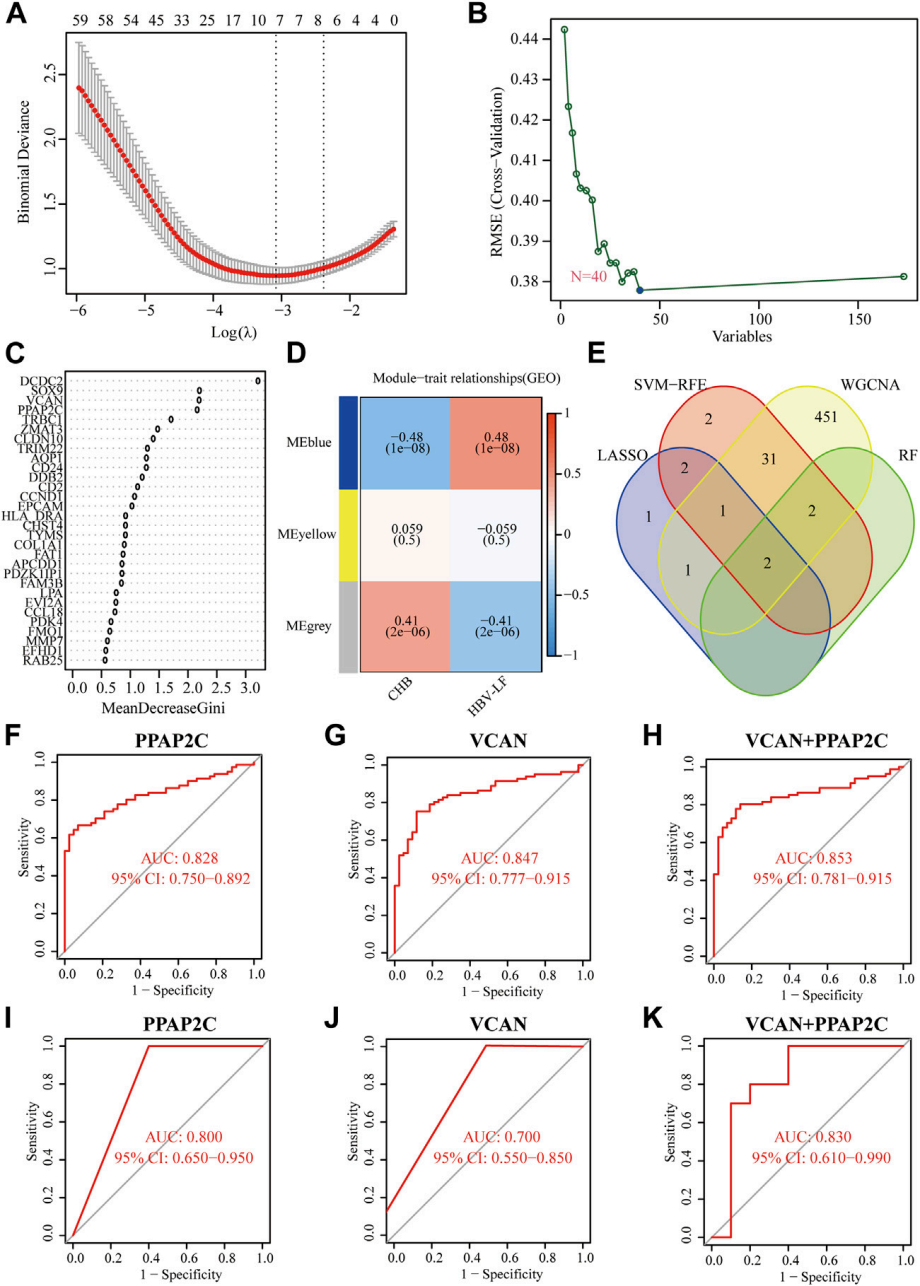
Identifying and verifying diagnostic biomarkers *via* the comprehensive strategy. **(A)** LASSO logistic regression. **(B)** SVM-RFE algorithm. **(C)** RF algorithm. **(D)** WGCNA analysis. **(E)** Venn diagram of the intersection of diagnostic biomarkers screened. **(F–H)** The ROC analysis of PPAP2C, VCAN, and PPAP2C + VCAN in the training set. **(I–K)** The ROC analysis of PPAP2C, VCAN, and PPAP2C + VCAN in the validation set.

To identify reliable diagnostic biomarkers from the DEGs, we integrated four algorithms, including LASSO, SVM-RFE, RF, and WGCNA. Seven genes were screened out as diagnostic biomarkers by using the LASSO regression (Figure 4A; Supplementary Table S2). Forty candidate genes were identified by the SVM-RFE algorithm (Figure 4B). The application of the RF algorithm identified four genes with good generalization performance as potential biomarkers (Figure 4C). Three co-expression modules were screened by WGCNA analysis. The correlation between these modules and HBV-LF was shown in the heat map, which indicated that the blue module was the most significant one, with a total of 488 genes (Figure 4D). Finally, two genes, namely, phosphatidic acid phosphatase type 2C (PPAP2C), and VCAN, were identified as the potential diagnostic biomarkers for HBV-LF based on the four algorithms (Figure 4E). ROC curve analyses were performed to clarify whether these two genes could distinguish HBV-LF from CHB samples. The results revealed that the AUC values of PPAP2C and VCAN were 0.828 (95% CI 0.750–0.892) and 0.847 (95% CI 0.777–0.915), respectively (Figures 4F,G). In the validation set, the AUC values of PPAP2C and VCAN were 0.840 (95% CI 0.630–1.000) and 0.740 (95% CI 0.500–0.960), respectively (Figures 4I,J). The AUC values of PPAP2C and VCAN were significantly higher than those of ALT, AST, and AST/ALT (Supplementary Figure S1). Furthermore, the combined AUC values of PPAP2C and VCAN reached 0.853 (95% CI 0.781–0.915) and 0.830 (95% CI 0.610–0.990) in the training and validation cohort, respectively (Figures 4H,K). These results indicated that PPAP2C and VCAN had excellent specificity and sensitivity for the diagnosis of HBV-LF.

**FIGURE 5:**
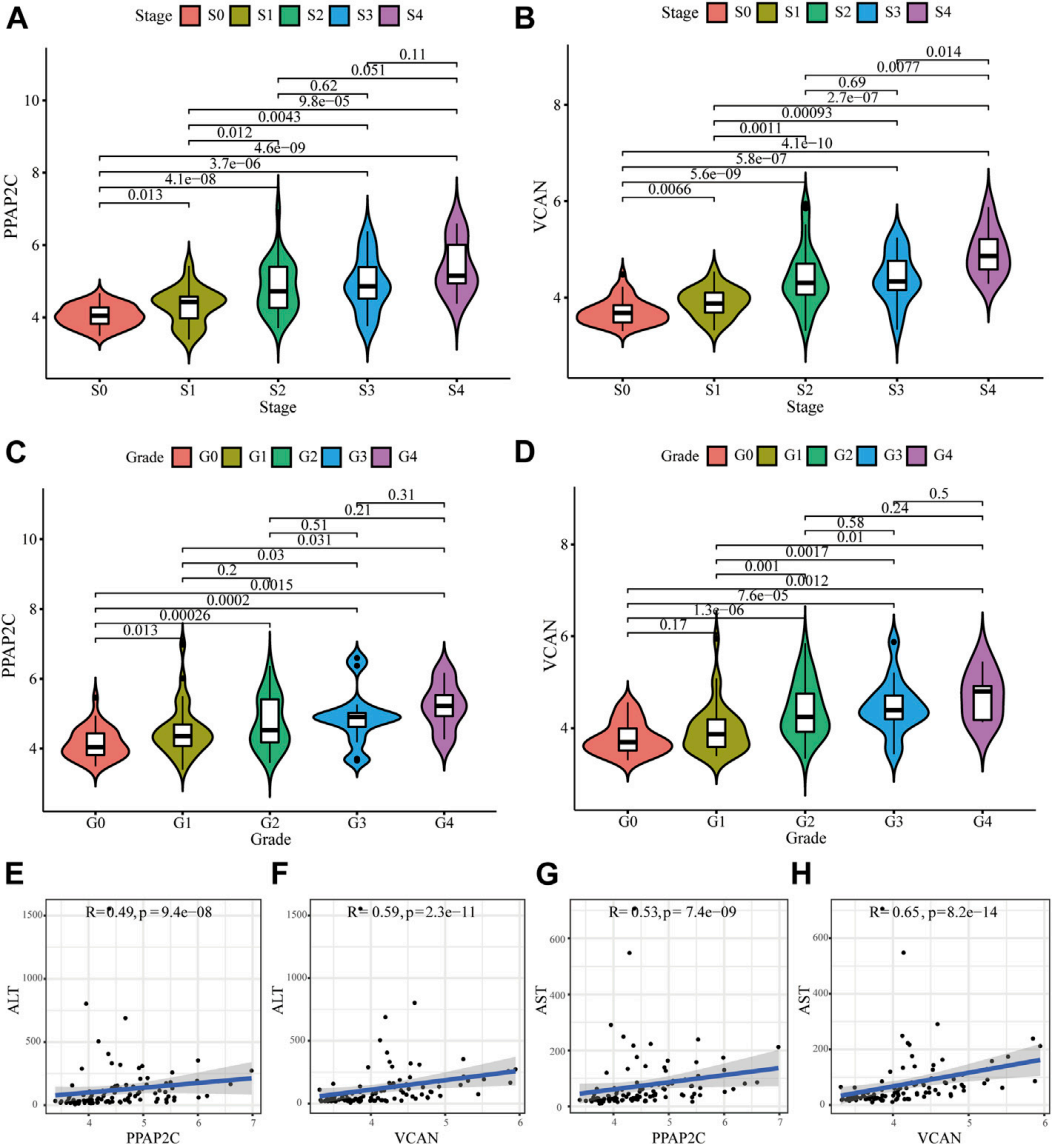
Relationships between the diagnostic biomarkers and clinicopathological features. **(A–D)** The expression level of PPAP2C and VCAN in different groups divided by clinical characteristics. **(E–H)** The correlation analysis between PPAP2C and VCAN and ALT, and AST.

We further evaluated the relationships between these two diagnostic biomarkers and disease progression. Generally, liver biopsy remains the gold standard for the diagnosis of LF, and the progress of LF can be evaluated according to histological fibrosis stages and inflammatory grades. The results depicted that as the histological fibrosis stages and inflammatory grades gradually increased, the expression levels of PPAP2C and VCAN also increased (Figures 5A,D). Additionally, two biochemical markers, ALT and AST, are often used to assess liver inflammation and function. As depicted in Figures 5E,H, the expression level of PPAP2C and VCAN were highly associated with ALT (R = 0.49, *p* = 9.4e−08, and R = 0.59, *p* = 2.3e−11, respectively) and AST (R = 0.53, *p* = 7.4e−09, and R = 0.65, *p* = 8.2e−14, respectively). The above results indicated that PPAP2C and VCAN were closely related to the progress of HBV-LF.

### Immune cell infiltration profile and correlation analysis

**FIGURE 6:**
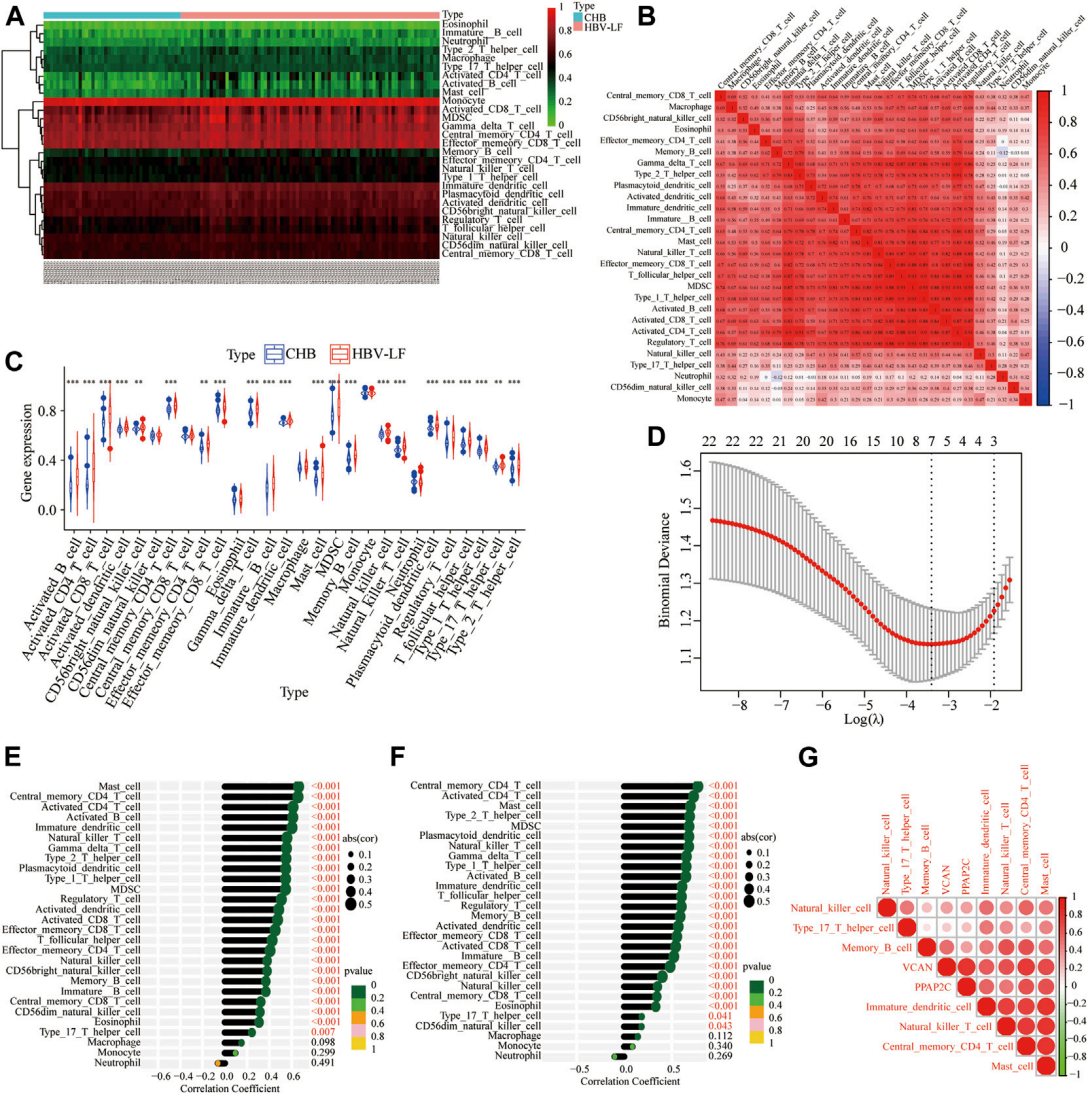
Immune cell infiltration pattern analyses of HBV-LF and CHB samples. **(A)** Heatmap of the immune landscape by ssGSEA algorithm. **(B)** Correlation heatmap of 28 immune cells. **(C)** Violin diagram of 28 immune cells. **(D)** LASSO analysis to screen the different infiltrates of immune cells. **(E–G)** Correlation between PPAP2C and VCAN and differential immune cells in HBV-LF.

To comprehensively understand the immune landscape in HBV-LF and CHB samples, we assessed the relative infiltration levels of 28 immune cell subsets using ssGSEA algorithm (Figure 6A). Significant positive correlations was found among almost all immune cells, whereas neutrophils were negatively associated with memory B cells (Figure 6B). As shown in Figure 6C, 22 types of immune cells aberrantly differed between HBV-LF and CHB samples. These included activated B, activated CD4 T, activated CD8 T, effector memory CD4 T, effector memory CD8 T, gamma delta T, natural killer T, regulatory T, T follicular helper, type 1 T helper, type 17 helper T, type 2 T helper, activated dendritic CD56 bright natural killer, immature B, immature dendritic, mast, MDSC, memory B, natural killer and plasmacytoid dendritic cells. Subsequently, seven significantly different types of immune cells were extracted using LASSO regression. These included immature dendritic, mast, memory B, natural killer, natural killer T cells, central memory CD4 T, and type 17 helper T cells (Figure 6D). Compared with CHB, the number of immune cells in the HBV-LF samples increased significantly.

Correlation analysis showed that PPAP2C and VCAN positively correlated with almost all immune cells, except macrophages, monocytes, and neutrophils (Figures 6E,F). Furthermore, we analyzed the correlation between the two diagnostic biomarkers and seven significantly different immune cells (Figure 6G). PPAP2C was positively correlated with central memory CD4 T cell (R = 0.64, *p* < 0.001), immature dendritic cell (R = 0.59, *p* < 0.001), mast cell (R = 0.68, *p* < 0.001) and natural killer T cell (R = 0.52, *p* < 0.001). While VCAV was positively correlated with central memory CD4 T cell (R = 0.79, *p* < 0.001), immature dendritic cell (R = 0.62, *p* < 0.001), mast cell (R = 0.71, *p* < 0.001), memory B cell (R = 0.55, *p* < 0.001) and natural killer T cell (R = 0.64, *p* < 0.001).

### Enrichment of similar/interactive genes of VCAN

**FIGURE 7:**
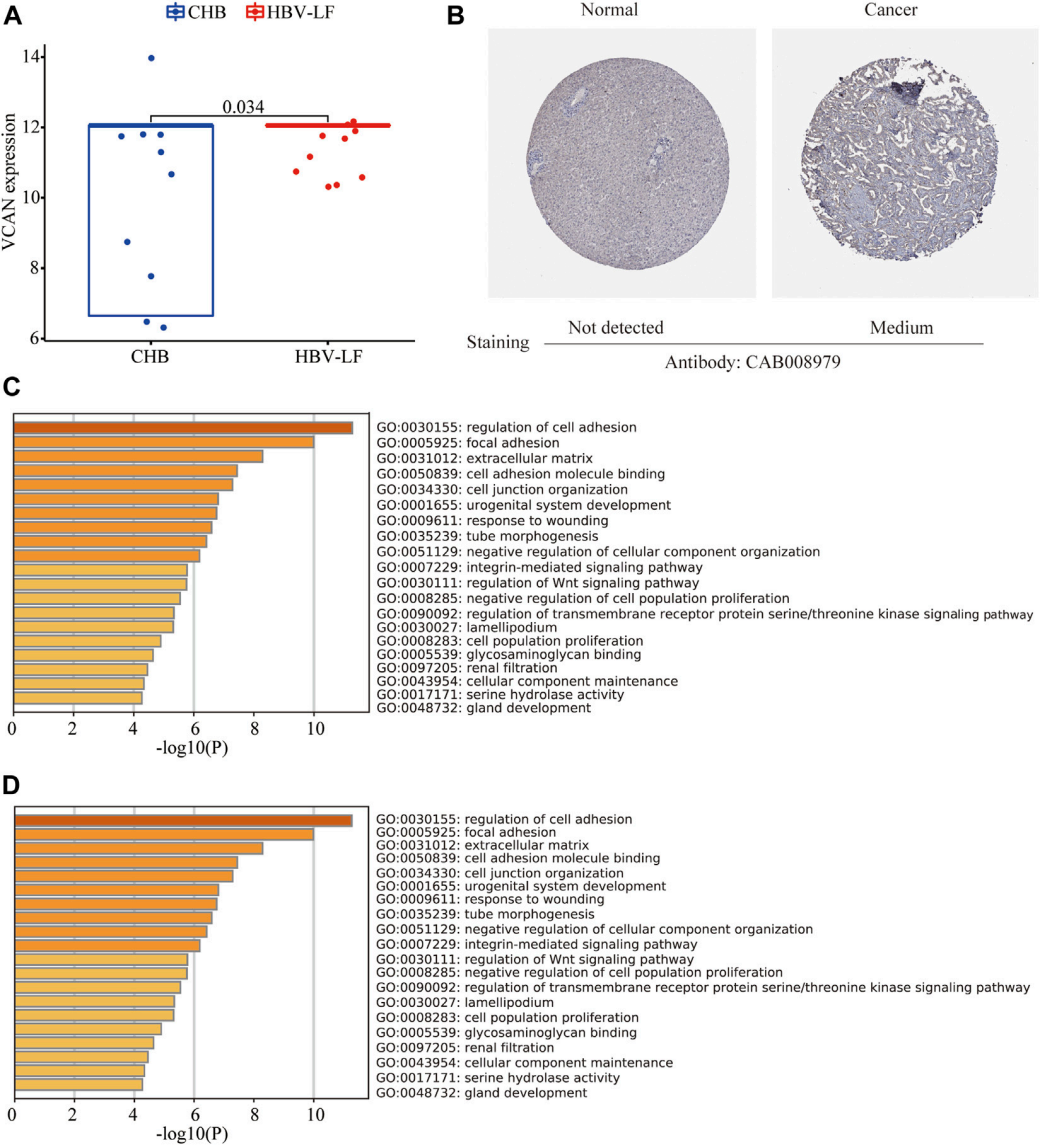
VCAN expression in HBV-LF and enrichment analysis of similar/interactive genes of VCAN. **(A)** The expression level of VCAN between the CHB and LF in the validation set. **(B)** The immunohistochemistry staining images of VCAN from the HPA. **(C)** GO analysis of similar/interactive genes of VCAN in Metascape database. **(D)** KEGG analysis of similar/interactive genes of VCAN in Metascape database.

VCAN is the main component of the ECM of early fibrogenesis. VCAN is related to the migration and proliferation of fibroblasts and promotes collagen deposition (Bukong et al., 2016). Therefore, we further compared the expression of VCAN between the CHB and LF patients, based on the transcriptomic profiles of peripheral blood mononuclear cells. Compared with CHB patients, the expression of VCAN in HBV-LF patients was significantly increased (Figure 7A). Several studies have found that VCAN is also present in the stroma of various types of cancers and is associated with cancer growth and invasion (Naboulsi et al., 2016). The immunohistochemical staining results of VCAN protein downloaded from the HPA database indicated moderate staining for VCAN protein in liver cancer, but no detectable expression in the normal liver tissue (Figure 7B).

Moreover, we identified 100 VCAN-related similar/interactive genes from the GEPIA database. Then, the Metascape website was applied to conduct an enrichment analysis of these 100 genes. GO analysis depicted that these similar/interactive genes were correlated with the regulation of cell adhesion, focal adhesion, and ECM (Figure 7C). KEGG analysis indicated that these similar/interacted genes mainly participated in the ECM-receptor interaction, cell adhesion molecules and transforming growth factor-beta (TGF-β signaling pathway (Figure 7D).

### Construction of the TF–VCAN–miRNA regulatory network

**FIGURE 8:**
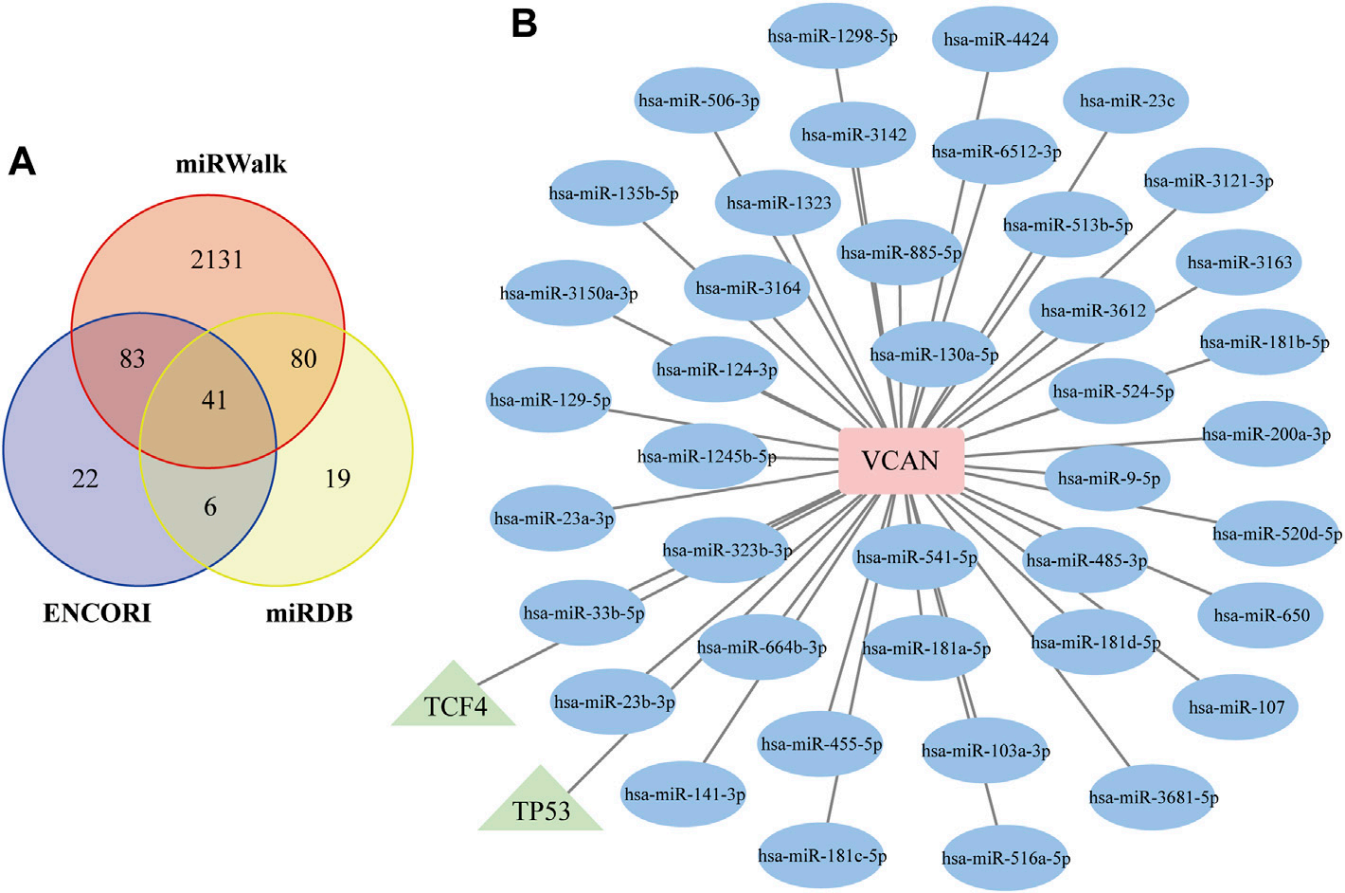
Construction of the TF–VCAN–miRNA Regulatory Network. **(A)** Venn diagram of the intersection of targeted miRNAs screened by ENCORI, miRWalk, and miRDB databases. **(B)** TF–VCAN–miRNA regulatory network. The square represents the gene, the circle represents miRNA, and the triangle represents TF.

To further assess the regulatory mechanisms of VCAN, we predicted the miRNAs and TFs that targeting VCAN. A total of 41 miRNAs targeting VCAN were identified through the intersection of miRNAs predicted by the ENCORI, miRWalk, and miRDB databases (Figure 8A). Interestingly, based on the TRUUST database, only two TFs [transcription factor 4 (TCF4) and tumor protein p53 (TP53)] that could regulate the expression of VCAN were screened out. Subsequently, we generated a regulatory network among VCAN, miRNAs, and TFs using Cytoscape software (Figure 8B).

## Discussion

CHB virus infection is a major public health problem that affecting 292 million individuals worldwide (Polaris Observatory, 2018). LF is an inevitable process by which CHB develops into HBV-related liver cancer (Schweitzer et al., 2015; Qi et al., 2016). Identification of effective diagnostic biomarkers is of great clinical significance for improving the prognosis of HBV-LF. In addition, HBV infection elicits various immune responses within the liver microenvironment, and the infiltration of immune cell is crucial for the development of HBV-LF (Zaki et al., 2022). Here, we attempted to screen diagnostic biomarkers for HBV-LF and explore the role exerted by the immune infiltration cells by using comprehensive and effective bioinformatics methods.

In this study, by comparing the matrix file of the HBV-LF and CHB samples, we identified 173 DEGs, of which 22 were downregulated and 151 were upregulated. To improve the accurate identification of HBV-LF-related diagnostic biomarkers, we integrated three different machine learning methods (LASSO, SVM-RFE, and RF algorithms) and WGCNA. Finally, PPAP2C and VCAN were identified as potential diagnostic biomarkers. ROC curve analysis further suggested that PPAP2C and VCAN exhibited high predictive accuracy for diagnosing HBV-LF, which were higher than that of ALT, AST, and AST/ALT. Moreover, PPAP2C and VCAN were positively associated with histological fibrosis stages, inflammatory grades, and two biochemical markers ALT and AST. These findings indicated that PPAP2C and VCAN are significantly correlated with the progression of HBV-LF.

PPAP2C (also known as PLPP2) is a triglyceride synthesis-related gene which encodes lipid phosphate phosphatase 2 (LPP2), a member of the phosphatidic acid phosphatase (PAP) family (Tang et al., 2015). Previous studies have reported that the increase in LPP2 expression in synchronized fibroblasts accelerates the entry of cells into the S phase (Morris et al., 2006). In addition, the expression of PPAP2/LPP2 is also upregulated in numerous carcinomas and sarcomas (Flanagan et al., 2009). However, there is no data on the role of PPAC2P in the development and progression of LF.

VCAN is a large, multi-domain chondroitin sulfate proteoglycan that upregulates inflammation in various diseases (Islam and Watanabe, 2020). The pathogenesis of LF is closely associated with persistent hepatic inflammation. Activation of hepatic stellate cells (HSCs) and collagen renewal are the main mechanisms of LF. Recent researches have shown that the expression of VCAN increases during the activation of HSCs and LF, while the proteolysis process occurs during the regression of LF (Bukong et al., 2016; Wight et al., 2020). Moreover, previous studies have suggested that LF is an inevitable stage in the progression of CHB to HBV-associated liver cancer, also known as the “hepatitis trilogy.” Interestingly, compared with paracancerous tissue, the expression level of VCAN was significantly upregulated in HCC tissue (Tanaka et al., 2018; Zhangyuan et al., 2020). In support, we also found that VCAN was significantly up-regulated in HBV-LF and HCC samples. Thus, VCAN may contribute to the progression of chronic liver disease to LF, cirrhosis and liver cancer. However, the specific mechanism by which VCAN determines the pathogenesis of HBV-LF requires further investigation. Given the enrichment analysis of VCAN-related similar/interacted genes, our results proposed the possibility that VCAN may promote LF through HSC-related mechanisms and the TGF-β signaling pathway. Taken together, these results showed that VCAN can be used as an effective diagnostic biomarker for HBV-LF patients.

Functional analysis of DEGs revealed that, except for the synthesis of ECM and the processes of anabolism and catabolism, they were also enriched in the immune response-related processes, which was consistent with previous studies (Yuan et al., 2019; Zaki et al., 2022). In this study, we further implemented the ssGSEA algorithm to evaluate the relative infiltration levels of immune cells. We found that the infiltration levels of 22 kinds of immune cells in the HBV-LF samples were significantly higher than those in CHB samples. Among them, seven kinds of immune cells were screened out by LASSO logistic regression, which included immature dendritic, mast, memory B, natural killer, natural killer T, type 17 helper T, and central memory CD4 T cell. Dendritic cells are key regulators of liver immunity, and abnormal dendritic cell phenotype can lead to the activation of T cells and HSCs, thus inducing a pathological fulminant environment and fibrogenesis (Xu et al., 2019). Mast cells are immune cells that are ubiquitous in all connective tissues and the mucosal environment. The pathogenicity of mast cells in LF is mainly realized by releasing a variety of mediators that directly interfere with the recruitment and activation of inflammatory cells, stimulate the proliferation of fibroblasts, promote synthesis or inhibit the degradation of ECM (Weiskirchen et al., 2019). Type 17 helper T cell is a subgroup of helper T cells that mainly secretes interleukin (IL)-17, a cytokine that promotes inflammation and fibrosis. It is currently evidenced that IL-17A can induce LF by directly activating HSCs in HBV patients and animal models of LF (Tan et al., 2013). Zhang et al. (2017) further confirmed that IL-17A inhibits hepatocyte autophagy through the IL-10/STAT3 signaling pathway and plays a pivotal role in the pathogenesis of LF. Moreover, natural killer, natural killer T, memory B, and central memory CD4 T cells have been demonstrated to be involved in the occurrence and development of LF (Wang and Yin, 2015; Huang et al., 2020; Zuluaga et al., 2020; Zuluaga et al., 2022). Therefore, our results were consistent with these previous reports, and our bioinformatics analysis further emphasized the important role of these immune cells in the pathogenesis of LF. In this study, we also found that PPAC2C and VCAN were significantly positively correlated with infiltrating immune cells. However, the molecular mechanisms and functions of immune cell infiltration in the development of HBV-LF are still urgent issues to be elucidated.

It should be pointed out that the present research still has some limitations. First, the study lacks clinically relevant information, including serum markers or indices. Therefore, we cannot compare the diagnostic efficacy of PPAC2C, VCAN, and conventional serological markers or indices. Second, the sample size is not sufficiently large and only from the GEO dataset. Third, ssGSEA is an algorithm to estimate immune cell infiltration based on gene expression, which requires further experimental verification.

## Conclusion

In the present study, we performed a comprehensive bioinformatics analysis of DEGs that may be involved in HBV-LF. VCAN may be an effective diagnostic biomarker of HBV-LF. This study also reveals that immune cell infiltration may contribute to the initiation and progression of HBV-LF. Our results may help to clarify the potential molecular mechanisms related to the occurrence, development, and prognosis of HBV-LF.

## Data Availability

The original contributions presented in the study are included in the article/Supplementary Material, further inquiries can be directed to the corresponding authors.
