# Supplementary figures and images for: Comprehensive bioinformatics and machine learning analysis identify VCAN as a novel biomarker of hepatitis B virus-related liver fibrosis
Source: Front Mol Biosci. 2022 Oct 7;9:1010160. doi: 10.3389/fmolb.2022.1010160 (PMC9585216; doi:10.3389/fmolb.2022.1010160)

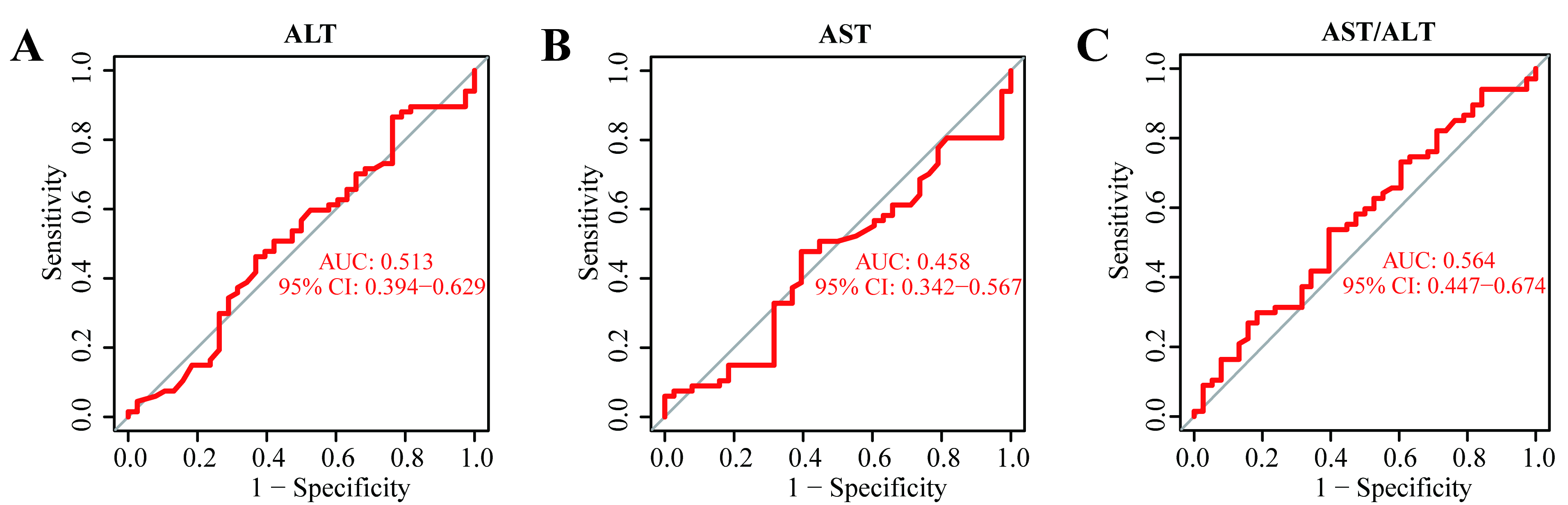

Supplement: Supplementary file 2 [file Image1.tif]
